# Seroepidemiology of canine leishmaniosis in Évora (southern Portugal): 20-year trends

**DOI:** 10.1186/1756-3305-6-100

**Published:** 2013-04-15

**Authors:** Henk DFH Schallig, Luís Cardoso, Saul J Semião-Santos

**Affiliations:** 1 Department of Parasitology, Koninklijk Instituut voor de Tropen (KIT), Royal Tropical Institute, Amsterdam, The Netherlands; 2 Department of Veterinary Sciences, School of Agrarian and Veterinary Sciences, University of Trás-os-Montes e Alto Douro, Vila Real, Portugal; 3 Parasite Disease Group, Instituto de Biologia Molecular e Celular (IBMC), Universidade do Porto, Oporto, Portugal; 4 Center for Diagnosis and Research in Leishmaniosis, Institute of Agrarian and Environmental Mediterranean Sciences (ICAAM), University of Évora, Evora, Portugal

**Keywords:** Canine Leishmaniosis, Direct Agglutination Test, Évora, Portugal, Seroprevalence

## Abstract

**Background:**

Canine leishmaniosis (CanL) is an endemic zoonosis in the southern regions of Europe. This paper reports the trend in CanL seroprevalence in the municipality of Évora (southern Portugal), where the disease is endemic, over a period of 20 years. The work comprises three different studies that were conducted in the years of 1990 (n = 3,614), 1999 (n = 3,563) and 2010 (n = 1,485 dogs). Blood samples were collected during the anti-rabies vaccination campaigns. Anti-*Leishmania* antibodies were detected with the direct agglutination test (DAT).

**Findings:**

The total percentages of DAT seropositive dogs were 3.9% (in 1990), 9.4% (in 1999) and 5.6% (in 2010). The overall seroprevalence was significantly higher in 1999 compared to 1990, but in 2010 a significant decrease was found in comparison with 1999. However, compared to 1990 the overall seroprevalence was still significantly higher in 2010. From 1990 to 2010 seroprevalence has switched from significantly lower to higher in the rural areas. Relatively few dogs showed clinical signs of overt disease (0.8% to 2.0%) with lymphadenopathy, onychogryphosis and skin involvement as most frequently observed. Gender associated differences in seroprevalence were not found, and most commonly seropositive dogs were working or stray animals. The mean age of seropositive dogs was significantly higher than seronegative dogs in all three sampling rounds.

**Conclusions:**

A high proportion of dogs, which are apparently healthy, yet seropositive, may remain an important factor in limiting the outcome of zoonotic leishmaniosis control efforts.

## Findings

Leishmaniosis, a disease caused by protozoan parasites of the genus *Leishmania,* is one of the major communicable diseases in the world and one of the most-important vector-borne parasitic diseases after malaria [1]. Dogs are the main domestic reservoir for human infection where zoonotic leishmaniosis is caused by *Leishmania infantum*[2]. Canine leishmaniosis (CanL) is a systemic chronic disease and clinical manifestations usually include lymphadenopathy, dermatitis, alopecia, cutaneous ulcerations, onychogryphosis, lameness, anorexia, weight loss, cachexia, ocular lesions, epistaxis, anaemia, diarrhoea, and renal failure [3]. Even when they receive treatment, severely affected dogs do not often survive the disease. Nevertheless, a significant proportion of infected animals remain asymptomatic [4], but these asymptomatic infected dogs may act as carriers of *L. infantum* and are capable of transmitting the parasite to the vector, the phlebotomine sand flies [5]. CanL is endemic in all countries of the Mediterranean basin, including the European countries Portugal, Spain, France, Italy, Greece, Croatia, Albania, Malta, and Cyprus. Emerging trends in the seroprevalence of CanL in many traditional leishmaniosis foci are being reported [4,6-8]. Possibly due to global warming, CanL is currently also being reported in foci outside the classical area of the disease in the Mediterranean countries [9-11]. For example, cases of autochthonous CanL have recently been reported from Hungary [12] and Germany [13].

In Portugal, there are four main CanL foci: the province of Trás-os-Montes e Alto Douro (in the north), the region of Lisbon (in the west), the province of Algarve (in the south) and the municipality of Évora (in the southern region of Alentejo). Previous studies conducted in Trás-os-Montes e Alto Douro have demonstrated that there is a considerable prevalence of CanL in northern Portugal with approximately 20% of dogs being seropositive [14,15]. In the context of emerging disease, the present study describes trends in seroprevalence of CanL over two decades in the municipality of Évora in southern Portugal (38° 34′ 17″ N, 07° 54′ 31″ W). Évora comprises 19 different administrative sections (parishes) of which eight are considered to be urban and the other rural, covering an area of 1,307.04 km^2^ with an average altitude of 300 m above sea level. The climate and vegetation are typically Mediterranean, with dry hot summers (32-35°C) and maximum rainfall in spring and autumn, and mild winters with temperatures rarely going below 5°C. The sand fly season runs from May to October: *Phlebotomus sergenti* is the most abundant species, followed by *Phlebotomus perniciosus*[16]. The known species of *Leishmania* is *L. infantum* MON-1 [17].

This study was approved by the University of Évora ethics committee for research in health sciences and well-being as complying with the Portuguese legislation for the protection of animals (Law no. 92/1995, from September the 12th). Dogs were surveyed during the yearly anti-rabies vaccination campaigns in 1990 (n = 3,614), 1999 (n = 3,563) and 2010 (n = 1,485). All animals were clinically examined by veterinary practitioners and considered as either apparently healthy or clinically suspect, respectively, when either none or at least one clinical sign or lesion compatible with CanL was noted. Blood samples were collected from the cephalic vein, and spotted into the middle of a filter paper allowed to air-dry and stored at -20°C until testing with the direct agglutination test (DAT) for titration of anti-*Leishmania* antibodies [18,19]. The samples analysed in 1990 and 1999 were tested by using a liquid DAT antigen prepared from *Leishmania donovani* promastigotes (strain 1-S) with a cut off titre of 1:320 [16]. The samples collected in 2010 were analysed with DAT based on freeze dried antigen, with a cut off titre of 1:400 [18,19]. Chi-square test compared proportions of positivity (no. of dogs found seropositive divided by the no. of dogs tested) related to the categorical dependent variables location, gender and ability. The exact binomial test was used to calculate confidence intervals (CI) for proportions, with a 95% confidence level. Differences between the ages of dogs were compared with the Mann–Whitney U test. Analyses were done with StatLib or SPSS 11.5 software for Windows. A probability (*p*) value < 0.05 was regarded as statistically significant [20].

The serology data obtained in 1990 have been previously reported [16], but again presented in the current paper to allow a comparison over a time period of 20 years. The total numbers of DAT seropositive dogs were 141 (3.9%) in 1990, 335 (9.4%) in 1999 and 84 (5.6%) in 2010. The overall seroprevalence was significantly higher in 1999 (9.4%) compared to 1990 (3.9%), but in 2010 a significant decrease in seroprevalence (5.6%) was found compared to 1999 (Table 1). Nevertheless, the overall seroprevalence was significantly higher in 2010 compared to 1990. From 1990 to 1999 the total increase in seroprevalence was simultaneous with significant increases in both the urban and rural areas. On the other hand, from 1999 to 2010 the decrease in seroprevalence was simultaneous with a significant decrease only in the urban area (Table 1). At last, when comparing the years 1990 and 2010, the increase in seroprevalence was simultaneous with a significant increase only in the rural area. A questionnaire conducted in Portugal in the year 2006 revealed that dog owners in urban areas had a significantly higher knowledge on CanL compared to the rural ones [21]. No data is available on the prophylactic measures applied to dogs from Évora, but it might be possible that preventatives [22], especially insecticides with a repellent effect, have increasingly been used on dogs from the urban area.

**Table 1:** Percentages of seropositive dogs found in urban or rural settings in Évora municipality

**Study area**	**Year 1990**		**Year 1999**		**Year 2010**	
	**% seropositive (No. dogs tested)**	**95% CI (%)**	**% seropositive (No. dogs tested)**	**95% CI (%)**	**% seropositive (No. dogs tested)**	**95% CI (%)**
Urban	4.9^a ^(1,524)	3.9–6.1	↑ 10.1 (1,661)	8.7-11.7	↓ 3.3^b ^(675)	2.1–4.9
Rural	3.2^a ^(2,090)	2.5–4.0	↑ 8.8 (1,902)	7.5-10.1	7.6^b ^(820)	5.8–9.6
Total	3.9 (3,614)	3.3–4.6	↑ 9.4 (3,563)	8.5-10.4	↓ 5.6 (1,495)	4.5–6.9

^a^, ^b^: statistically significant difference between areas; ↑↓: statistically significant difference to the previous year, for the same area.

Out of the 141 seropositive dogs in 1990, 113 (80.1%) were apparently healthy and 28 (19.9%) were considered clinically suspect (0.8% of the total study population in 1990). Similar observations were made in 1999 and 2010, when 263 healthy dogs (78.5%) and 72 (21.5%) suspect dogs (2.0% of the total study population) were DAT positive (out of the total of 335 animals) and 71 (80.1%) healthy and 13 (9.9%) suspect dogs (0.9% of the total study population) were seropositive (out of 84), respectively. From 1990 to 1999 there was a significant increase in the percentages of suspect dogs among the total populations; and from 1999 to 2010 there was a significant decrease. However, differences between the proportions of clinically suspect dogs among the seropositive ones were not statistically significant when comparing the years 1990–1999 or 1999–2010. The percentage of clinically suspect dogs among the total populations seems to have followed that of the seropositivity. Among every 5.0 (year 1990), 4.7 (year 1999) or 6.5 (year 2010) seropositive dogs there was one animal clinically suspect of CanL. Lymphadenopathy (69.2-89.3%), followed by onychogryphosis (53.2-59.7%) and skin involvement (38.9-46.4%), were the most frequently observed clinical manifestations in the present study and are among those most commonly found [3,4]. The number of dogs with overt disease is thus relatively low, but at a comparable level found in other studies in the Iberian Peninsula [4,14].

Gender associated differences between male and female seroprevalences were not found in this study (Table 2) and are in line with previous observations [4,14,23], but in contrast to some other studies in which a higher prevalence was observed in males [24]. With respect to ability or occupation of the dogs, it was noted that the highest levels of seropositivity were found among stray dogs, but the majority of seropositive dogs in absolute numbers were mainly working animals, including guard and hunting dogs (Table 2). An association between age and seroprevalence was also observed (Table 3), in all three rounds of sample collection, and confirmed previous findings that dogs of older age are at a higher risk of being seropositive [4,8,15].

**Table 2:** Percentages of seropositive dogs in relation to gender and ability

	**Year 1990**	**Year 1999**	**Year 2010**
	**% seropositive (No. dogs tested)**	**% seropositive (No. dogs tested)**	**% seropositive (No. dogs tested)**
Gender			
Female	3.4 (1,926)	↑ 9.3 (1,837)	↓ 6.2 (796)
Male	4.5 (1,688)	↑ 9.5 (1,726)	↓ 5.0 (699)
Ability			
Guard	3.6 (1,483)	↑ 7.9* (1,702)	↓ 3.7* (854)
Hunting	3.8 (992)	↑ 13.7* (774)	11.0* (227)
Pet	4.7 (656)	5.9* (612)	3.7 (246)
Shepherd	3.4 (351)	↑ 12.1 (298)	↓ 5.0 (119)
Stray	11.5 (61)	17.9* (117)	↑ 41.4* (29)
ND	0.0 (71)	3.3 (60)	0.0 (20)
Total	3.9 (3,614)	↑ 9.4 (3,563)	↓ 5.6 (1,495)

↑↓: statistically significant difference to the previous year; *: statistically significant difference to the total, for the same year; ND: no data on ability available.

**Table 3:** Relationship between age of dogs and seropositivity

**DAT**	**Year 1990**		**Year 1999**		**Year 2010**	
	**Mean age (± SD) – years**	**No. dogs**	**Mean age (± SD) – years**	**No. dogs**	**Mean age (± SD) – years**	**No. dogs**
Seropositive	7.04 (± 2.71)^a^	141	6.96 (± 2.98)^b^	335	6.57 (± 3.37)^c^	84
Seronegative	4.71 (± 2.40)^a^	3,473	↑5.26 (± 2.76)^b^	3,227	5.34 (± 2.81)^c^	1,411
Total	4.80 (± 2.46)	3,614	↑5.42 (± 2.82)	3,562*	5.41 (± 2.86)	1,495

DAT: direct agglutination test; SD: standard deviation; ^a^, ^b^, ^c^: statistical significant difference between seropositive and seronegative; ↑: statistical significant difference to the previous year; *: age was not determined for one dog.

The findings of the presented surveys reveal that a considerable number of dogs from Évora are seropositive for CanL. A peak in seropositivity was observed in 1999 and there is a decline in the number of positive cases in 2010. It should however be noted that the total number of dogs sampled in 2010 was much lower (< 50%) compared to previous sample rounds. This could be due to the fact that people, perhaps influenced by the current economic crisis, are becoming reluctant to have their dogs anti-rabies-vaccinated and tested, and may dispose of animals with ill health (in 2010 only 13 animals with clinical signs of CanL were found in the present study). This may bias, i.e. underestimate, the true seroprevalence in the region. The reported seroprevalences are in a comparable range to those reported in other studies from Portugal [25,26]. Also, in other countries in the Mediterranean basin a rather wide range of seroprevalence is being reported [4,6,8].

## Conclusions

In conclusion, there is a high proportion of dogs that are seropositive for CanL in the municipality of Évora in Portugal but that appear as clinically healthy. If in analogy to human infections [27] where asymptomatic cases are thought to be also in the majority, with an estimated ratio of > 100 asymptomatic individuals per each clinical case, the number of infected dogs could be enormous. Indeed, it is difficult to provide an actual number of infected dogs, but it has been estimated based on seroprevalence studies that 2.5 million dogs from Italy, France, Spain and Portugal are infected [2]. It remains a concern that so many animals are possibly infected with *L. infantum* and that they may transmit the parasite either to other dogs or humans [3,28]. Therefore, control efforts should remain focussed on canines, but also the human population in these regions should be better monitored too.

## Abbreviations

CanL: Canine leishmaniosis; DAT: Direct agglutination test.

## Competing interests

The authors declare that they have no competing interests.

## Authors’ contributions

HDFHS performed DAT (2010) and data analysis and wrote the manuscript. LC performed data analysis, tabulation and revision of the manuscript. SJSS organized the collection of canine samples and data and performed DAT analysis (1990 and 1999). All authors read and approved the final manuscript.
